# Development and evaluation of nomograms for predicting osteoarthritis progression based on MRI cartilage parameters: data from the FNIH OA biomarkers Consortium

**DOI:** 10.1186/s12880-023-01001-w

**Published:** 2023-03-27

**Authors:** Chunbo Deng, Yingwei Sun, Zhan Zhang, Xun Ma, Xueyong Liu, Fenghua Zhou

**Affiliations:** 1 Department of Orthopedics, Shengjing Hospital of China Medical University, Shenyang, Liaoning Province China; 2 Department of Orthopedics, Central Hospital of Shenyang Medical College, Shenyang, Liaoning Province China; 3 Department of Radiology, Shengjing Hospital of China Medical University, Shenyang, Liaoning Province China; 4 Department of Radiology, Affiliated Hospital of Liaoning University of Traditional Chinese Medicine, Shenyang, Liaoning Province China; 5 Department of Rehabilitation, Shengjing Hospital of China Medical University, No.16, Puhe Street, Shenyang North New Area, Shenyang, Liaoning Province 110134 China

**Keywords:** Osteoarthritis, Cartilage, Nomogram, Magnetic resonance imaging

## Abstract

**Background:**

Osteoarthritis (OA) is a leading cause of disability worldwide. However, the existing methods for evaluating OA patients do not provide enough comprehensive information to make reliable predictions of OA progression. This retrospective study aimed to develop prediction nomograms based on MRI cartilage that can predict disease progression of OA.

**Methods:**

A total of 600 subjects with mild-to-moderate osteoarthritis from the Foundation for National Institute of Health (FNIH) project of osteoarthritis initiative (OAI). The MRI cartilage parameters of the knee at baseline were measured, and the changes in cartilage parameters at 12- and 24-month follow-up were calculated. The least absolute shrinkage and selection operator (LASSO) regression analysis was used to extract the valuable characteristic parameters at different time points including cartilage thickness, cartilage volume, subchondral bone exposure area and uniform cartilage thickness in different sub regions of the knee, and the MRI cartilage parameters score0, scoreΔ12, and scoreΔ24 at baseline, 12 months, and 24 months were constructed. ScoreΔ12, and scoreΔ24 represent changes between 12 M vs. baseline, and 24 M vs. baseline, respectively. Logistic regression analysis was used to construct the nomogram0, nomogramΔ12, and nomogramΔ24, including MRI-based score and risk factors. The area under curve (AUC) was used to evaluate the differentiation of nomograms in disease progression and subgroup analysis. The calibration curve and Hosmer-Lemeshow (H-L) test were used to verify the calibration of the nomograms. Clinical usefulness of each prediction nomogram was verified by decision curve analysis (DCA). The nomograms with predictive efficacy were analyzed by secondary analysis. Internal verification was assessed using bootstrapping validation.

**Results:**

Each nomogram included cartilage score, KL grade, WOMAC pain score, WOMAC disability score, and minimum joint space width. The AUC of nomogram0, nomogramΔ12, and nomogramΔ24 in predicing the progression of radiology and pain were 0.69, 0.64, and 0.71, respectively. All three nomograms had good calibration. Analysis by DCA showed that the clinical effectiveness of nomogramΔ24 was higher than others. Secondary analysis showed that nomogram0 and nomogramΔ24 were more capable of predicting OA radiologic progression than pain progression.

**Conclusion:**

Nomograms based on MRI cartilage change were useful for predicting the progression of mild to moderate OA.

**Supplementary Information:**

The online version contains supplementary material available at 10.1186/s12880-023-01001-w.

## Background

Knee Osteoarthritis (KOA) is the most common progressive multifactorial joint disease [1]. The global total prevalence of knee osteoarthritis in people aged 40 and over is 22.9% [2]. With the aging population, the number of patients with OA is increasing [3]. KOA has a significant impact on health-related quality of life (HRQOL) and imposes a huge burden on individuals and the economy [4–6]. Although KOA is classically described as slowly progressing, KOA is heterogeneous, at least 10% of patients with OA have a rapid disease progression that can lead to the need for total joint replacement [7, 8]. The current drugs can only alleviate the symptoms of KOA, and the development of disease-modified OA drugs (DMOADs) that prevent or reduce the progression of joint tissue deterioration lags behind other arthritis diseases. However, current clinical diagnostic procedures do not adequately fulfil the need of clinicians to help patients reduce their risk of disease progression or the need of the health-care industry to develop effective new DMOADs [8]. Advances in osteoarthritis diagnostics, prevention, and treatment will have a major impact on patients and society.

Magnetic resonance imaging (MRI) is considered the most comprehensive imaging modality for knee osteoarthritis assessment [9]. Although KOA affects all tissues in the joint, cartilage is still the main research target for predicting disease progression and treat diseases. MRI has become a widely used and recognized method to measure the structural changes of knee osteoarthritis (KOA), because it has been proved to be an effective and repeatable technology is more sensitive to detecting changes in KOA than X-rays. Although X-ray is safe and economical, it remains the most important imaging method for diagnosing KOA clinically [10]. Whole Organ MRI Score (WORMS) and MRI OA Knee Score (MOAKS) systems are used to assess the degree of knee cartilage damage [11, 12]. However, it is currently unknown to what extent such cartilage damage is associated with quantitative structural outcomes, such as longitudinal change in 3D cartilage thickness obtained from cartilage segmentation [13]. Therefore, Eckstein et al. performed a regional analysis of the femoral-tibial cartilage using the novel technique and explored the relationship between baseline MRI-detected femoral-tibial cartilage damage and longitudinal changes in cartilage thickness and knee function. In another study with KL grading of 1–3, semi-quantitative cartilage damage at baseline was associated with quantitative loss of cartilage thickness at follow-up [14]. The MRI cartilage parameter contains centrally performed measurements of cartilage thickness, cartilage surface morphology, cartilage volume, and the percentage of subchondral bone denuded area in the corresponding area [15–17]. Eckstein et al. found the loss of medial tibiofemoral cartilage thickness was closely related to the progression of radiological OA [18]. Wirth et al. found that the loss of medial cartilage can predict the progress of radiological OA [19]. The thinner thickness of the central medial tibia at baseline and the 12-month change of the medial central femur from baseline were associated with extensive full-thickness cartilage loss [20]. Meanwhile, with the application of artificial intelligence in the field of MRI, many studies have established OA prediction models based on MRI cartilage semiquantitative scores or MRI cartilage morphology [21, 22]. However, existing methods for evaluating patients with OA do not provide sufficiently comprehensive information to make reliable predictions or prognosis [8].

Nomograms are an important component of modern medical decision making [23]. A well-constructed nomogram for answering a focused question, when properly interpreted and applied, can be of great value to the clinicians and patients [24]. With the ability to generate an individual numerical probability of a clinical event by integrating diverse prognostic and determinant variables, nomograms fulfill our desire for biologically and clinically integrated models and our drive towards personalized medicine [24]. The development and progression of KOA are related to several medical, biological and environmental risk factors [25]. Therefore, the nomogram may play the same role in KOA as it has in the field of oncology. To the best of our knowledge, there are only a few published articles on applying nomogram in KOA [26, 27]. Wu et al. screened clinical factors and knee MRI parameters to construct nomogram, so as to quantitatively predict the risk of knee replacement in patients with early osteoarthritis during the follow-up period [26]. In earlier studies, we used 3D bone shape to construct nomograms to predict the radiological progress of OA, the AUC of nomogram is 0.75 [27].

The aim of this study is, to develop and evaluate a nomogram, using quantitative cartilage morphology parameters at baseline and the corresponding changes in cartilage parameters from baseline to follow-up measurements in the 12th month (12 M) and 24thmonth (24 M), and clinical risk factors, from the Foundation for the FNIH OA Biomarkers Consortium, to predict pain progression and radiographic progression in KOA. We envisaged that a predictive nomogram would be useful to aid in clinical decision making.

## Materials and methods

### Subjects

This sted data and images from FNIH.The FNIH is a nested case-control study involving 600 subjects with KL level 1 to 3 (https://nda.nih.gov/oai/study_ documentatinon.html). Details of the study design have been previously published [28]. There were 194 subjects who had radiographic progression and pain progression simultaneously in the case group. Radiographic progression was defined as a reduction in the minimumudy width of the medial tibiofemoral joint space ≥ 0.7 mm from baseline to 24, 36, or 48 months. Pain progression was defined as a persistent increase of ≥ 9 points (0-100 normalized score) using the Western Ontario and McMaster Universities Arthritis Index (WOMAC) pain subscale at ≥ 2 time points from baseline to 24, 36 or 48 months the 24- to 60-month pain assessment. Persistence required a pain increase of ≥ 9 points at ≥ 2 timepoints from the 24-month to 60-month pain assessment. Knees were also excluded if there were not enough follow-up time points after the first increase in WOMAC pain data above the threshold to determine whether the increase was persistent. The 406 subjects in the control group did not meet the previously mentioned conditions. The control group was further divided into three subgroups. Among them, 200 subjects had no progression, 103 had only pain progression, and 103 had only progression on imaging.

### MR image acquisition and measurement of cartilage parameters

The MRI acquisition protocol of the OAI was described previously [29]. MRI acquisition was performed using a 3 T MRI system (Trio, Siemens Healthcare, Erlangen, Germany) at the four OAI clinical sites(The Ohio State University, University of Maryland, School of Medicine,University,Memorial Hospital of Rhode Island). The coronal 2-dimensional intermediate-weighted (IW) turbo spin-echo (TSE), sagittal 3-dimensional (3D) dual-echo at steady-state (DESS), coronal and axial multiplanar reformations of the 3D DESS and sagittal IW fat-suppressed (fs) TSE sequences were used to measure cartilage thickness, cartilage denudation, cartilage volume within the subregion [16, 30–32].

The FNIH released a set of parameters related to MRI knee cartilage measured by Eckstein [15, 18]. Based on several previous studies, the medial and lateral tibia and femoral articular cartilage were divided into 8 subregions, with 5 further tibial subregions (anterior, posterior, central, internal, and external) and 3 femoral weight-bearing subregions (central area, central medial area, and central lateral area). Each subregion includes quantitative cartilage parameters such as cartilage thickness, cartilage volume, subchondral bone exposure area, and cartilage thickness uniformity [30].

### MRI feature parameter extraction and establishment of cartilage morphology parameters score

We used the least absolute shrinkage and selection operator (LASSO) logistic regression algorithm to screen the MRI cartilage morphology parameters at baseline and the changes in cartilage parameters at follow-up 12 M and 24 M, respectively. All cartilage parameters are from FNIH(Osteoarthritis Biomarkers Consortium FNIH Project: Measurement of Cartilage Volume/Thickness by Chondrometrics). The three different cartilage morphology score (score0, scoreΔ12, and scoreΔ24) were respectively constructed using the cartilage morphology parameters or the changes at different times, which was calculated as a linear combination of selected features that were weighted by their respective LASSO coefficients [33].

### MRI cartilage parameters nomogram construction

Backward stepwise multivariate logistic regression analysis was used to screen the clinical risk factors at baseline and the MRI score at baseline, 12 M, and 24 M in order to construct the nomogram (named nomogram0, nomogramΔ12, and nomogramΔ24,respectively). The likelihood ratio test using the Akaike information criterion was used as the stopping rule for backward stepwise logistic regression analysis. The variance inflation factor (VIF) was applied to diagnose col-linearity in variable logistic regression. Clinical risk factors included age, sex, body mass index (BMI), the minimum medial compartment joint space width in the medial tibiofemoral region (MCMJSW), Kellgren-Lawrence (KL) grade, Western Ontario and McMaster Universities Arthritis Index WOMAC pain score (WOMKP), and WOMAC disability score (WOMADL).

### Performance assessment of MRI nomogram

The AUC and ACC were used to evaluate the discrimination of the nomogram, and the calibration curve and Hosmer-Lemeshow (H-L) test were used to evaluate the calibration of the nomogram. The p value of H-L test is greater than 0.05, which proves the perfect consistency between the predicted and the observed values.The internal validation was carried out by bootstrapping method (1000 bootstrap resamples) to reduce the over-fitting deviation. The clinical usefulness of the nomogram was verified by decision curve analysis (DCA) [34, 35]. Additionally, net reclassification improvement (NRI) and relative integrated discrimination improvement (IDI) were analyzed to evaluate nomogram improvements compared with the nomogram only including the clinical covariates (nomogram-CO). We further evaluated the predictive value of nomogram0 and nomogramΔ24 among different subgroups.

### Statistical analysis

The expectation-maximization method was used to interpolate the missing data. Cartilage morphology parameters were Z-normalized to facilitate comparison of parameters (Z = x-µ/σ). Continuous variables are expressed as mean ± standard deviation (SD). Data that were normally distributed were tested using independent-samples t-test, and non-normally distributed variables were tested using the Mann-Whitney *U* test. Categorical variables were represented by numbers and percentage, and categorical variables were tested using chi-square analysis and Fisher exact test. All tests were two-sided with p < 0.05 indicating statistical significance. A variance inflation factor (VIF) was leveraged to analyze the co-linearity of various factors in the logistic regression analysis, and VIF > 10 considered indicative of multi-collinearity. Data analysis was conducted using IBM SPSS, version 22.0, Empower (R) (http://www.empowerstats.com, X & Y solutions, Inc., Boston MA) and R 4.0.2 (http://www.Rproject.org).

## Results

### Clinical characteristics at baseline

The subjects in the case group and the control group were frequency matched for their baseline characteristics including sex, age, BMI, WOMKP, and WOMADL, but there was a significant difference in KL grade at baseline ( Supplementary Table 1).

### Selection of MRI characteristic parameters and construction of cartilage score

**Fig. 1:**
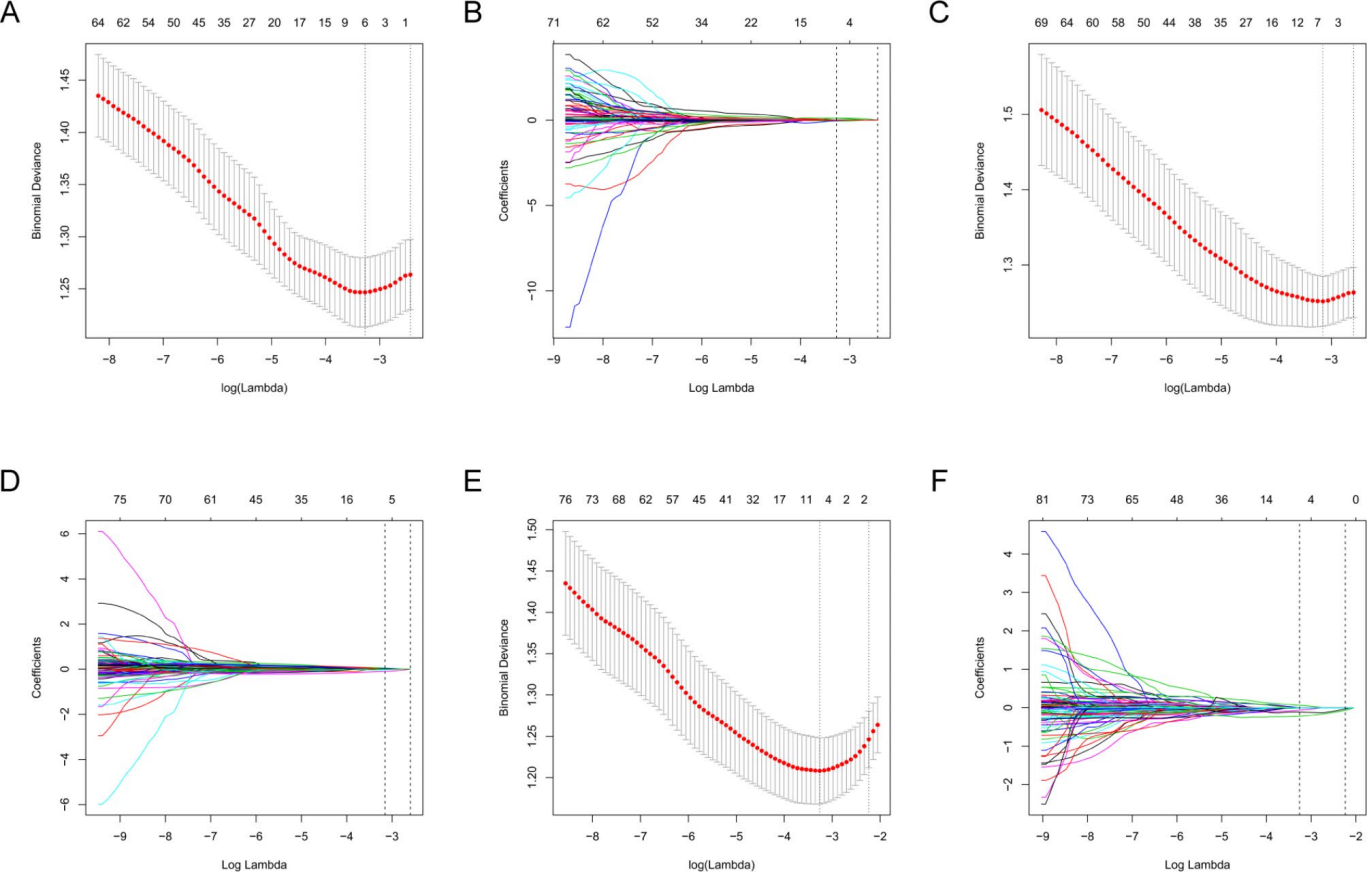
Selection of cartilage morphology parameters features using LASSO binary logistic regression nomogram.Tuning parameter (λ) selection in the LASSO nomogram was used in the 10-fold cross-validation via minimum criteria and the 1 standard error of the minimum criteria (the 1-SE criteria). The binomial deviance was plotted versus log(λ). (**A**) Dotted vertical lines were drawn at the optimal λ values, the λ value of 0.0379, with log(λ)–3.272, was the chosen via minimum criteria at baseline. (**B** ) LASSO coefficient profiles of the 92 cartilage parameters. The dotted vertical line was plotted at the λ value of 0.0379, resulting in 6 parameters at baseline. (**C**) A λ value of 0.0427, with log (λ)–3.154, was chosen via the minimum criteria at 12 M. (**D**) LASSO coefficient profiles of the 92 cartilage parameters. The dotted vertical line was plotted at the λ value of 0.0427 via minimum criteria, resulting in 6 parameters at 12 M. (**E**) A λ value of 0.0384 with log (λ)–3.26 was chosen via minimum criteria at 24 M. (**F**) LASSO coefficient profiles of the 92 change cartilage parameters over 24 M.The dotted vertical line was plotted at the λ value of 0.1068 via minimum criteria, resulting in 5 parameters at 24 M

At the 12 M of follow-up, the MRI data of 18 subjects were missing, and at the 24 M of follow-up, the MRI data of 1 subject was missing. We used the expectation-maximization method to interpolate the missing data. A formula was generated using a linear combination of selected features that were weighted by their respective LASSO coefficients; the formula was then used to calculate a risk score for each patient to reflect the progression of KOA. Score0 included 6 cartilage parameters at baseline, scoreΔ12 included 6 changes of cartilage parameters at 12 M, and ScoreΔ24 included 5 changes of cartilage parameters via minimum criteria at 24 M, respectively (Table 1; Fig. 1).

**Table 1:** The Selected MRI cartilage parameters of score and their corresponding coefficients

	The cartilage parameters of score at different time	coefficients
score0	coefficient of variation of cartilage thickness - medial tibia	0.107674849
	The percent of area of subchondral bone denuded of cartilage - anterior medial tibia (%)	0.000946295
	The percent of area of subchondral bone denuded of cartilage - central medial femur (%)	0.116403371
	minimum cartilage thickness - central medial femur (mm)	-0.045685031
	mean cartilage thickness - central medial femur (mm)	-0.033840691
	% area of subchondral bone denuded of cartilage -external central medial (%)	0.005973847
scoreΔ12	The change of minimum cartilage thickness -center medial tibia	-0.103293043
	The change percent of subchondral bone denuded of cartilage - center medial tibia(%)	0.006184577
	The change percent of area of subchondral bone denuded of cartilage - anterior medial tibia (%)	0.051352482
	The change percent of area of subchondral bone denuded of cartilage - central medial femur (%)	0.034920672
	The chang mean cartilage thickness - central medial femur (mm)	-0.021555888
	The change percent of area of subchondral bone denuded of cartilage - central medial femur (%)	0.005259763
scoreΔ24	The change of minimum cartilage thickness of central medial tibia (mm)	-0.062594417
	The change of area of cartilage surface of central medial femur(cm^2)	-0.006184622
	The change percent of area of subchondral bone denuded of cartilage - central medial femur(%)	0.065893315
	The change of mean cartilage thickness of medial tibia-femur compartment (mm)	-0.113420248
	The change of mean cartilage thickness of central medial tibia-femur compartment (mm)	-0.220041334

### Construction and performance assessment of chondral nomogram

**Fig. 2:**
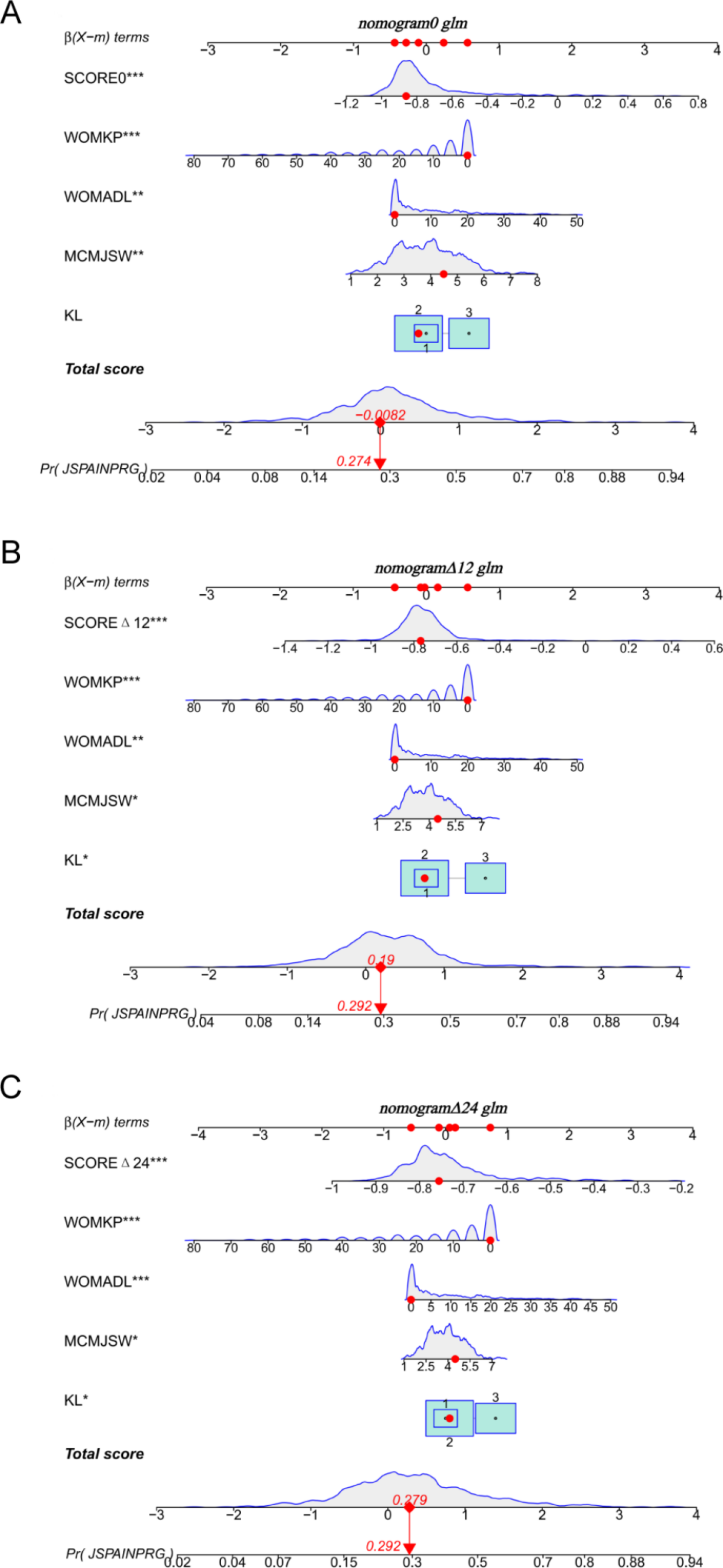
Construction of nomograms based on MRI cartilage parameters for predicting the progression of OA. The distribution and total point number of predictive variables overlap along the nomogram scales. The box plot shows the categorical variables (e.g., KL grade ), with the box size indicating percentage. The density plot shows the distribution of continuous variables (e.g., WOMKP, MCMJSW, WOMADL, score0, scoreΔ12, scoreΔ24 and total score). The definite value of each red point corresponds to the scale of the variable’s axis (β(χ-m) term). The observation values overlap at the total score axis. A straight line is drawn through this point and downward extending to the risk axis, and the point of intersection with the risk axis represents the occurrence probability of the progression of KOA. (**A**) nomogram0. (**B**) nomogramΔ12. (**C**) nomogramΔ24

**Fig. 3:**
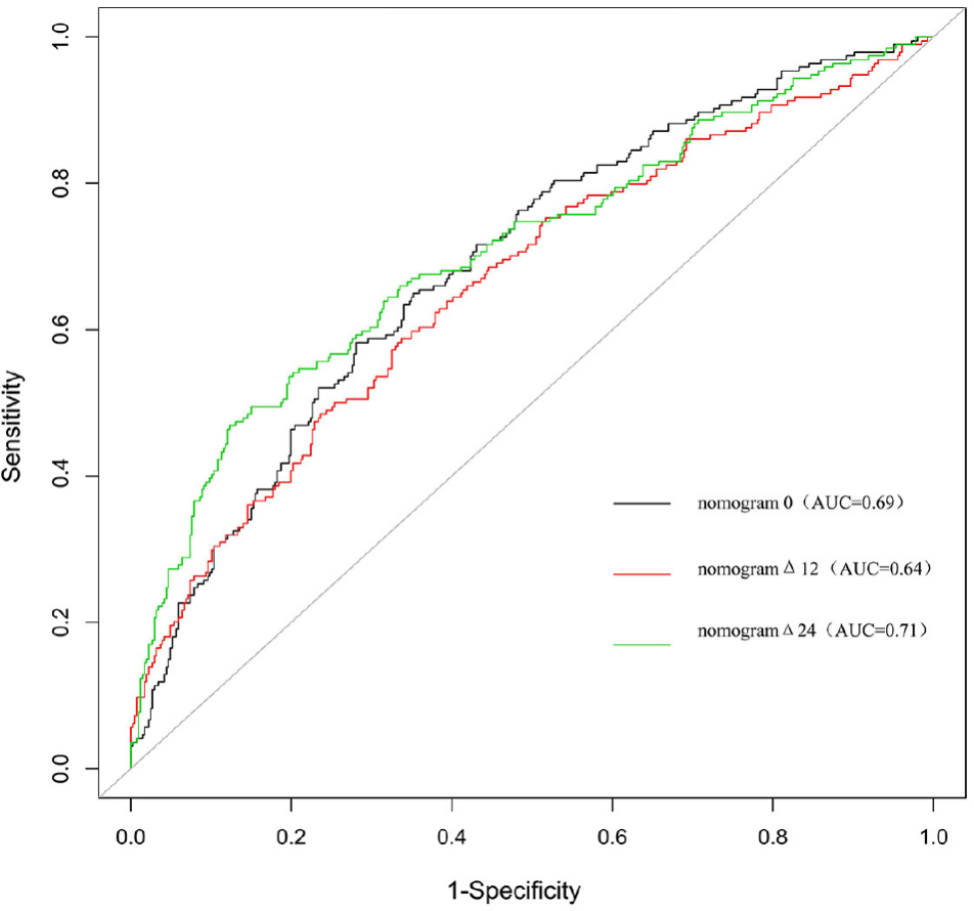
Receiver curve analyses of the nomograms to compare the predictive performance at different times.The AUC of nomogramΔ24 was higher than nomogramΔ12 and nomogram0

**Fig. 4:**
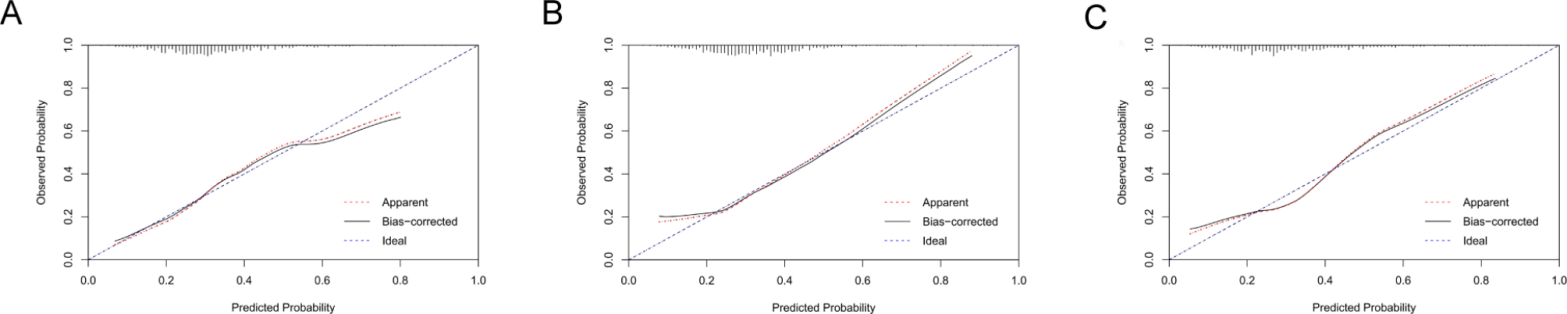
The calibration curve illustrates the calibration of the nomogram in terms of the agreement between the predicted risk of OA and the observed outcomes of OA. The 45° solid grey line represents a perfect prediction, and the dotted red line represents the predictive performance of the nomogram. The dotted line has a closer fit to the solid line, which indicates better predictive accuracy of the nomogram. (**A**) nomogram0. (**B**) nomogramΔ12. (**C**) nomogramΔ24

**Fig. 5:**
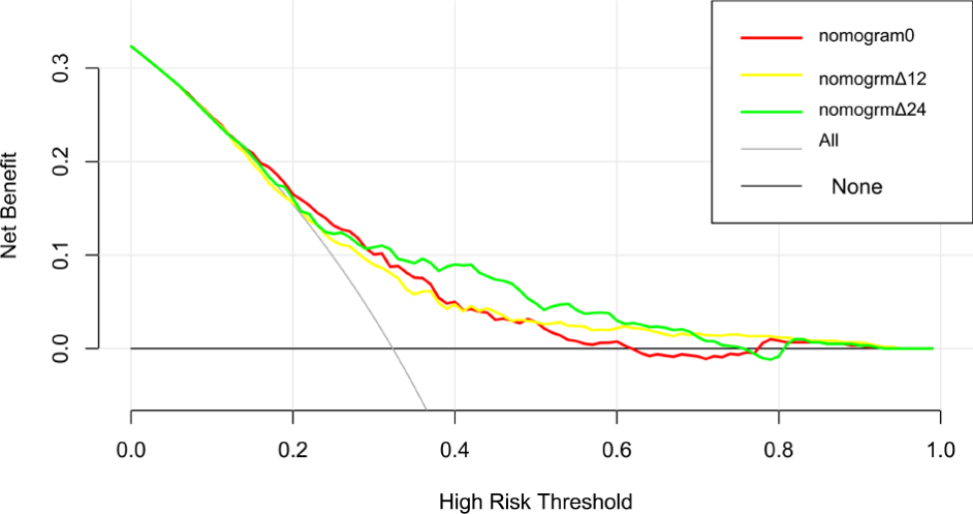
Decision curve analysis for nomograms predicted the radiological and pain progression of KOA. The Y axis shows the net benefit. The x axis of DCA is the threshold of the predicted probability using the nomogram to classify subjects with progression and subjects without progression. The gray line represents the hypothesis that all subjects had OA progression; the black line represents the hypothesis that none of the subjects had any progression of OA. The DCA illustrates that all models were useful between threshold probabilities of 4-70%,and the net benefit of the modelΔ24 was better than the other

The three nomograms established were nomogram0, nomogramΔ12, and nomogramΔ24, including MRI cartilage score and KL, WOMPK, WOMADL, and MCMJSW, respectively, at different times (Table 2; Fig. 2). The VIF of all predictive factors in the nomogram were all less than 5, indicating that there was no collinearity among variables. The AUC of nomogram Δ24 (0.71, 95% CI [confidence interval]: 0.66 to 0.79) was higher than the AUC of nomogram 0 (0.69, 95% CI 0.63 to 0.72), p>0.05. The AUC of nomogram Δ24 (0.71, 95% CI: 0.66 to 0.79) was higher than the AUC of nomogram 0 (0.60, 95% CI 0.60 to 0.70), p < 0.05. The ACC of nomogram0, nomogramΔ12 and nomogramΔ24 are 0.68, 0.66 and 0.77 respectively( Fig. 3). Compared with nomogram-CO, nomogramΔ24 had the highest performances in NRI and IDI. The NRI of nomogram0 was 0.0878 (95% CI -0.0061 to 0.0816, p > 0.05), and the IDI of nomogram0 was 0.0505 (95% CI 0.0307–0.0704, p < 0.05). The NRI of nomogramΔ12 was 0.0685 (95% CI − 0.0202 to 0.1572, p > 0.05), and the IDI of nomogramΔ12 was 0.043 (95% CI 0.0228–0.0631, p < 0.05). The NRI of nomogramΔ24 was 0.3083 (95% CI 0.1897–0.4268, p < 0.05), and the IDI of nomogramΔ24 was 0.091 (95% CI 0.0648–0.1173, p < 0.017).The P values of H-L test of nomogram0, nomogramΔ12, and nomogramΔ24 were 0.92, 0.35, and 0.67, respectively, indicating that the three nomograms had good calibration. The calibration curve showed that the predicted results of nomogram were consistent with the actual results of OA progression (Fig. 4). DCA shows that all nomograms are clinically effective at range of roughly 4 to 100%.Whereas the net benefit of nomogramΔ24 is better than other nomograms when the threshold probability is approximately 30% to70% (Fig. 5).

**Table 2:** Risk factors for the progression of OA at baseline, 12 M, and 24 M by multiple logistic regression

	nomogram0		nomogramΔ12		nomogramlΔ24	
Intercept and variable	0R	95%CI	0R	95%CI	0R	95%CI
Intercept	0.65	0.18 to 2.29	1.37	0.31 to 6.06	0.53	0.15 to 1.83
BL MCMJSW	1.44	1.15 to 1.81	1.27	1.02 to 1.58	1.29	1.03 to 1.63
XRKL2	0.9	0.51 to 1.59	0.98	0.56 to 1.72	1.07	0.59 to 1.92
XRKL3	1.8	0.86 to 3.77	2.24	1.09 to 4.63	2.17	1.02 to 4.60
BL WOMACKP	0.95	0.93 to 0.98	0.95	0.93 to 0.98	0.94	0.92 to 0.97
BL WOMADL	1.05	1.02 to 1.09	1.05	1.02 to 1.08	1.06	1.03 to 1.10
Score0	11.22	4.52 to 27.85				
ScoreΔ12			18.76	5.12 to 68.88		
ScoreΔ24					5.88	3.54 to 9.77

### Secondary analysis

The secondary analysis was used to further evaluate the predictive power of nomogram0 and nomogramΔ24, which were highly distinguished in primary analysis (Table 3). nomogram0 and nomogramΔ24 have the best discrimination in distinguishing OA progression (both radiological and pain progression) and non-progression, with an AUC of 0.74 and 0.77, respectively. nomogram0 and nomogramΔ24 have the lowest ability to distinguish between the pain-only progression group and non-progression group, with an AUC of 0.63 and 0.62, respectively.

**Table 3:** The seconday analysis of Nomgram0, NomogramΔ24

secondary analysis	Nomogram0		NomogramΔ24	
	AUC	ACC	AUC	ACC
Rad only progressor vs. non-progressor	0.68	0.59	0.77	0.75
Pain only progressor vs. non-progressor	0.63	0.70	0.62	0.72
Rad + pain progressor vs. non-progressor	0.74	0.70	0.77	0.75
All Progressors (Rad or Pain) vs. non-Progressors	0.67	0.58	0.71	0.62
Rad Progressors vs. Rad Non-Progressors	0.68	0.66	0.76	0.71
Pain Progressors vs. Pain Non-Progressors	0.68	0.64	0.67	0.64

## Discussion

In the current study, we screened the feature parameters of MRI cartilage at different time points using LASSO logistic regression, developed and validated nomograms to predict the progression of KOA. The nomogram0 and nomogramΔ24 achieved good performance, the ROC showed good discriminatory ability in predicting the progression of KOA. Therefore, Our study shows that nomogram0 and nomogramΔ24 can predict the progression of KOA.

In our study, we calculated the score of the MRI cartilage parameters. The selected cartilage parameters all come from medial compartment of tibiofemoral joint, which is consistent with previous results [18]. They concluded that the loss of medial tibial and femoral cartilage during 24 months of follow-up was the main risk factor for the progression of OA. However, there are also studies that have yielded inconsistent results. It has been reported that the change in lateral cartilage volume of the knee is closely related to the progression of medial tibiofemoral [36]. In the study of Hunter Group’s analysis of FNIH-related data to determine the best combination of imaging and biochemical biomarkers used to predict the progress of knee osteoarthritis (OA), the central medial femoral thickness and the change of the thickness of the central medial femur during the follow-up of 24 months can be used to predict the progress of OA, which is consistent with this study. This contradiction may be the result of different imaging methods, different image analysis techniques, different stages of OA. Our study shows that nomogram0 and nomogramΔ24 can predict the progression of KOA.

The results of our study suggest that nomogram0 and nomogramΔ24 have high discrimination ability, whereas nomogramΔ12 has low discrimination ability. By using backward stepwise selection with the AIC in logistic regression modelling, we identified the following 6 cartilage parameters as having the strongest associations with the progression of KOA. The degree of cartilage injury at baseline are believed to be one of the causes for the progression of OA at the end of the follow-up period. Our results are consistent with other studies. Lower baseline central medial tibial was associated with incident widespread full-thickness cartilage loss in KOA [20]. Cartilage defects of the knee are believed to be the cause of the development of OA, resulting in pain and dysfunction [37]. Frobell et al. found that OA subchondral bone exposure already existed in the early stage of OA (KL = 1 and KL = 2). With an increase in the severity of the disease, subchondral bone exposure becomes greater, the denuded areas of the subchondral bone and local cartilage loss was significantly associated with greater KL grades [38]. At the same time, recent studies suggest that the area of the cartilage defect has a specific spatial distribution in the knee joint, and OA cartilage denudation is limited to the bone center of the medial tibial and femoral joint [39]. With the loss of cartilage, the subchondral bone marrow in the defect area was also damaged, which further aggravates the progression of OA. Our study is similar to this study. At the baseline, the cartilage damage area is concentrated in the central area of the medial tibia and the central area of the medial femur [40]. A previous study supported the analysis of the uniformity of cartilage thickness rather than the average ROI thickness enhances the sensitivity to detect OA-related differences between knees [41]. Cartilage parameters involved in the construction of nomogramΔ24 reflect the loss of medial tibial and femoral articular cartilage thickness during the follow-up period, indicating that decrease of medial cartilage thickness is a risk factor for the progression of KOA.

Recent studies have focused on the measurement of anatomically defined cartilage subregions to clarify the spatial distribution of articular cartilage changes during the progression of OA.The change rate of cartilage morphology in the central subregion of the tibiofemoral was greater than that in the whole cartilage area, and the reduction of cartilage thickness was positively correlated with the OARSI score of joint space narrowing. The higher the OARSI score, the greater the decrease in cartilage thickness [42]. The degree of cartilage loss during the follow-up period was positively correlated with the exposure area of cartilage at baseline. The standardized response mean (SRM) of cartilage loss secondary to cartilage loss in non-exposed areas was − 0.25, whereas the SRM of cartilage loss in severe-exposed areas was − 1 [43].In conclusion, we believe that MRI cartilage parameters can correctly reflect the baseline cartilage status and the changes of knee cartilage during follow-up.

The nomogram has a user-friendly digital interface, higher ACC, and easier-to-understand prognosis, which can help health care professionals to make better clinical decisions. As a result, nomograms have been widely used in oncologic research [44]. In this study, the AUC of nomogram0 and nomogramΔ24 were 0.69 and 0.71, respectively. There are relatively few studies on the diagnosis or prediction of OA by nomogram. Zhang et al. applied the nomograph model to predict the severity of KOA through non-imaging parameters, which can intuitively identify patients with severe KOA, with AUC of 0.802 [45].

The control group included the no-progression subgroup, the pain progression subgroup, and the radiographic progression subgroup; other studies confirmed that this grouping reduced the predictive ability of the study parameters [46]. Normogram0 showed the highest degree of differentiation between the case group and the non-progressive subgroup, with an AUC of 0.75. The nomogramΔ24 provided the most discriminating ability when distinguishing the case group from the non-progression subgroup, or the radiographic-only progression subgroup from the non-progression subgroup. The nomogramΔ24 achieved an AUC of 0.77 in subgroup analysis. The two nomograms have the lowest ability to predict the pain-only progression subgroup and the non-progression subgroup.The etiology of pain in patients with knee OA is complex and multi-factorial. Lin et al. constructed nomogram based on MRI radiology and clinical variables, and achieved good predictive effect and accuracy in identifying OA and improving knee pain in patients with OA. This proof-of-concept study provides a promising method for predicting clinically significant results [47].

Some studies have found that there may be a process of cartilage edema and hypertrophy in the early stages of OA. These results suggest that OA progression is not one-way cartilage loss, but may be bidirectional-cartilage thinning and cartilage thickening-in the early stages of the lesion [48]. The KL grades of the included subjects in this study were 1 to 3. During the follow-up period, there were bidirectional changes in cartilage thickness during different stages of progression, and this complex pathological process may limit the predictive ability of the nomogram.

In addition to the study design [49], this investigation has a few other limitations. This study used bootstraps for internal validation. Although this method is no less than the internal verification of random grouping [50], the lack of external validation reduces the credibility of the nomogram.

Therefore, a large, multicenter sample is needed to verify the nomogram. Also, the variables screened by LASSO regression are inconsistent and have a lack of continuity at different time points, which is determined by the LASSO regression algorithm. Although statistical studies can prove the correlation between different scores and indirectly prove the correlation between different parameters, previous studies often judge the effect of a variable on the progression of OA or predict the progression of OA by observing the time-dependent concentration of a variable. Therefore, in this study, whether the selection of variables at different time points can really represent the pathological changes of articular cartilage needs further histologic verification. Furthermore, we only used MRI morphologic cartilage parameters were used to construct a prediction nomogram. Because of the complexity of OA progression, it may be necessary to combine biomarkers, cartilage morphology parameters, and the severity of OA to build a nomogram that better distinguishes OA progression.The cohort study design itself has certain limitations. Cartilage change was defined as the change from the baseline to 24 months of follow-up, while the progression of OA was assessed from baseline to 24, 36, or 48 months. Therefore, instead of having an absolute predictive relationship, there were some overlapping periods in the study.

## Conclusions

This study builds a predictive nomogram based on MRI quantitative cartilage parameters of KOA, combined with clinical risk factors. The results of this study suggest that this nomogram can predict the progress of mild-to-moderate KOA, and may have particular value in aiding health care professionals with clinical decision-making regarding KOA.

## Electronic supplementary material

Below is the link to the electronic supplementary material.

Supplementary Material 1

## Data Availability

All data are available from the OAI of the FNIH (https://data-archive.nimh.nih.gov/oai/), which is publicly available.

## List of abbreviations

OAI: Osteoarthritis initiative
FNIH: The Foundation for the National Institutes of Health
KL: Kellgren-Lawrence
AUC: The areas under curve
minJSW: Minimum joint space width
WOMKP: WOMAC pain score
WOMADL: WOMAC function score
LRT: likelihood ratio test
AIC: Akaike information criterion
VIF: variance inflation factor
DCA: The decision curve analysis
BMLs: Bone marrow lesions
NRI: net reclassification improvement
IDI: integrated discrimination improvement
