# Supplementary material for: Development and evaluation of nomograms for predicting osteoarthritis progression based on MRI cartilage parameters: data from the FNIH OA biomarkers Consortium
Source: BMC Med Imaging. 2023 Mar 27;23:43. doi: 10.1186/s12880-023-01001-w (PMC10045658; doi:10.1186/s12880-023-01001-w)
Supplement: Supplementary file 1 — Supplementary Material 1 [file 12880_2023_1001_MOESM1_ESM.docx]

| **Supplementary Table 1.**  Baseline characteristics of study subjects | | | |
| --- | --- | --- | --- |
|  | Control group  (n=406) | Case group  (n=194) | P-value |
| BMI | 30.71 ± 4.79 | 30.75 ± 4.77 | 0.991 |
| Age(years ) | 61.31 ± 8.92 | 62.04 ± 8.80 | 0.397 |
| BL MCMJSW | 3.85 ± 1.06 | 3.79 ± 1.39 | 0.183 |
| BL WOMADL | 8.68 ± 11.19 | 8.61 ± 9.41 | 0.311 |
| BL WOMACKP | 13.00 ± 16.66 | 10.15 ± 12.98 | 0.268 |
| XRKL |  |  | 0.017^#^ |
| 1 | 51 (12.56%) | 24 (12.37%) |  |
| 2 | 222 (54.68%) | 84 (43.30%) |  |
| 3 | 133 (32.76%) | 86 (44.33%) |  |
| sex |  |  | 0.463 |
| 1 (male) | 163 (40.15%) | 84 (43.30%) |  |
| 2 (female） | 243 (59.85%) | 110 (56.70%) |  |
